# THIRTY KILOGRAMS GIANT RETROPERITONEAL TERATOMA: CASE REPORT

**DOI:** 10.1590/0102-6720201600020016

**Published:** 2016

**Authors:** James SKINOVSKY, Fernanda Keiko TSUMANUMA, Marcos Fabiano SIGWALT, Flávio PANEGALLI-FILHO, Adriana Mitie KAWAKUBO, Bruna Gimenes ROLIM, Luciana Andrade de GODOY

**Affiliations:** Hospital da Cruz Vermelha and Universidade Positivo (Red Cross University Hospital and Positivo University), Curitiba, PR, Brazil.

**Keywords:** Tumor, teratoma, Retroperitoneal neoplasms

## INTRODUCTION

Teratomas are composed of somatic cells from two or more germ layers (ectoderm, mesoderm or endoderm)^8^. Although the child's age being the most affected, in adults it occurs at different locations, such as mediastinum, sacrococcix, retroperitoneum and more often in the gonads^7^ ^,^ ^13^. Retroperitoneal teratomas in adults are rare, representing only 1-11% of all primary tumors in that anatomic region^9^, generally are benign and asymptomatic in the first stages. However when symptoms occur, they are typically due to their size, presenting with abdominal distension and a palpable mass^12^. Diagnosis can be made by ultrasound, that can identify solid or cystic components, computerized tomography and magnetic resonance imaging, which are both superior than ultrasound to evaluate tumoral extention and relation to adjacent organs^2^ ^,^ ^4^ ^,^ ^5^ ^,^ ^12^ ^,^ ^13^. Angiography can be used to detect and evaluate the blood supply. In this article, it is presented a case of a giant retroperitoneal treated with surgical resection.

## CASE REPORT

**FIGURE 1:**
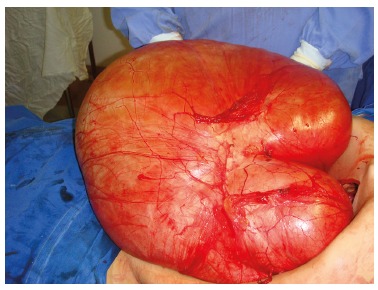
Surgically resected giant retroperitoneal tumor weighing approximately 30 kilograms

**FIGURE 2:**
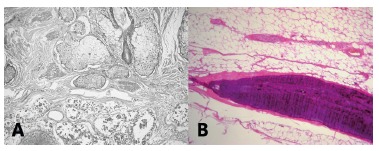
A) Elements from the cyst wall: cutaneous epidermis, dermis with sebaceous glands and apocrine sweat glands, adipose tissue; B) fragment of mineralized bone tissue (Courtesy: Doctor Lisiane Cristine, Citolab, Curitiba, PR, Brazil

A 42-year-old male was suffering from an insidious abdominal distention for the last 13 years, that was more remarkable in the initial three years. There was no fever, abdominal pain, or bowel complaints. He denied smoking or drinking abuse. There was not any kind of disease in patient's past or family medical history. He had been treated with spironolactone years before, with no previous investigation, and it was suspended by the occurrence of gynecomastia. On admission, he was clinically in good condition, and presenting an important abdominal distention without tenderness, and bowel sounds preserved. The rest of the examination was unremarkable. Admission laboratory tests showed no abnormalities. An abdominal computerized tomography revealed a mass occupying all regions in the abdominal cavity, showing no apparent origin. The patient underwent exploratory laparotomy that showed a mass weighing approximately 30 kilograms (Figure 1), whose origin was in the retroperitoneum completely displacing the left kidney to the right iliac fossa. The patient did well post-operatively and is currently assymptomatic and has been followed as an outpatient. Histopathological analisys demonstrated heterogeneous contents, with predominant cystic formations and other unctuous and soft components, and the diagnosis of mature teratoma was confirmed by microscopy (Figure 2).

## DISCUSSION

Histologically, teratomas are classified as mature or immature, and the mature type, which was described in this case, has the most common occurrence^3^ ^,^ ^7^ ^,^ ^8^. Mature teratomas are usually cystic, benign and have well-differentiated elements, resembling adult tissues, as can be observed in the case as well.

Migratory property of the germ cells may explain the ocurrence of teratomas in extragonadal sites. The retroperitoneal account for approximately 5% of teratomas and are responsible for less than 10% of the retroperitoneal neoplasms. In the retroperitoneal space, teratomas have a predilection for the upper pole of the kidney and are frequently located on the left side^12^ ^,^ ^13^. In this case, the tumor was located in retroperitoneum, completely displacing the left kidney to the right iliac fossa due to its large volume.

This type of tumor can affect both children and adults, exhibiting different behavior between the two groups. With higher incidence in the retroperitoneal region in children, less than 20% of patients develop these tumors by the age of thirty, though the patient in this case was in the fourth decade of life at diagnosis. Considering gender, teratomas at a retroperitoneal location affect approximately twice as many women than men^1^ ^,^ ^8^ ^,^ ^13^.

Most of the patients are asymptomatic and when the tumor compresses adjacent structures, due to its growth, it can bring pain, bloating, nausea and vomiting. Malignant teratomas tend to progress more quickly and occur more frequently in adults than in children, with incidences of 26% and 10%, respectively^8^ ^,^ ^13^. In this case, the only complaint the patient had was an insidious abdominal distention, no other complaints, as would be expected due to the size of the tumor found and the compression of adjacent structures. Computerized tomography was the imaging method used for the diagnosis and to plan the surgical procedure, which is, according to the literature^2^ ^,^ ^4^ ^,^ ^5^ ^,^ ^12^ ^,^ ^13^, one of the best methods, like magnetic resonance imaging, compared to ultrasound. The final diagnosis was made with the histopathological analysis after surgery.

Pinson et al^11^, on a long-term study, showed that complete resection is associated with improved survival rates for primary retroperitoneal tumors in general. A disease-free survival is related to complete resection because of the risk of malignant teratoma or carcinoma to sarcoma. The patient underwent complete excision of the tumor and is currently asymptomatic and being followed as an outpatient.Testicular ultrasound is necessary to rule out a coexisting testicular germ cell tumor in male patients, because 50% of men presenting a retroperitoneal tumor also have testicular carcinoma in situ, a precursor for testicular germ cell tumors^6^.
